# Emerging Artificial Neuron Devices for Probabilistic Computing

**DOI:** 10.3389/fnins.2021.717947

**Published:** 2021-08-06

**Authors:** Zong-xiao Li, Xiao-ying Geng, Jingrui Wang, Fei Zhuge

**Affiliations:** ^1^Ningbo Institute of Materials Technology and Engineering, Chinese Academy of Sciences, Ningbo, China; ^2^Center of Materials Science and Optoelectronics Engineering, University of Chinese Academy of Sciences, Beijing, China; ^3^School of Materials Science and Engineering, Southwest University of Science and Technology, Mianyang, China; ^4^School of Electronic and Information Engineering, Ningbo University of Technology, Ningbo, China; ^5^Center for Excellence in Brain Science and Intelligence Technology, Chinese Academy of Sciences, Shanghai, China

**Keywords:** brain-inspired computing, artificial neurons, stochastic neurons, memristive devices, stochastic electronics

## Abstract

In recent decades, artificial intelligence has been successively employed in the fields of finance, commerce, and other industries. However, imitating high-level brain functions, such as imagination and inference, pose several challenges as they are relevant to a particular type of noise in a biological neuron network. Probabilistic computing algorithms based on restricted Boltzmann machine and Bayesian inference that use silicon electronics have progressed significantly in terms of mimicking probabilistic inference. However, the quasi-random noise generated from additional circuits or algorithms presents a major challenge for silicon electronics to realize the true stochasticity of biological neuron systems. Artificial neurons based on emerging devices, such as memristors and ferroelectric field-effect transistors with inherent stochasticity can produce uncertain non-linear output spikes, which may be the key to make machine learning closer to the human brain. In this article, we present a comprehensive review of the recent advances in the emerging stochastic artificial neurons (SANs) in terms of probabilistic computing. We briefly introduce the biological neurons, neuron models, and silicon neurons before presenting the detailed working mechanisms of various SANs. Finally, the merits and demerits of silicon-based and emerging neurons are discussed, and the outlook for SANs is presented.

## Introduction

Chaos is generally undesirable for artificial intelligence architectures, long-term chaotic fluctuations in human brain waves exhibit significant functions in biological neural networks. High-level brain functions, such as memory recall and inference rely on the presence of certain types of noises, which are the functions desired to be mimicked in artificial neural networks (ANNs). Microscopically, the noise is generated by stochastic neuronal dynamics. Several complex phenomena, such as ionic conductance noise, chaotic motion of charge carriers caused by thermal noise, interneuron morphological variabilities, and synaptic background input noise (Faisal et al., 2008) have been considered as the source of stochastic neuronal behavior. Probabilistic computing based on stochastic neural networks is considered a feasible method of mimicking the inference function. This is because the response variability of cortical neurons observed in electrophysiological recordings has been well-explained in terms of probabilistic computation (Shadlen and Newsome, 1998). To date, stochastic computing algorithms based on restricted Boltzmann machine (Jordan et al., 2019) and Bayesian inference (Sountsov and Miller, 2015) have exhibited remarkable advantages in edge detection (Joe and Kim, 2019), traffic prediction (Sun X. et al., 2020), and the complex prediction of protein functions (Zou et al., 2017). However, the existing stochastic neural networks remain at quasi-stochastic states and are accelerated by the central processing unit or graphic processing unit. Moreover, the dedicated stochastic electronic circuits are in the early stages of development (Hamilton et al., 2014) and require more electric components. To sufficiently imitate the noise observed in brains, the hardware implementation for probabilistic computing should rely on the true stochastic sources of noise, particularly in terms of the inherent random nature to reduce the complexity of circuits. Additionally, mimicking the physical structure of biological neural systems can improve the operability and transplantability of computations (Pickett and Stanley Williams, 2013; Thalmeier et al., 2016; Kumar et al., 2017a).

Owing to the similarity in architectures based on synapses and neurons of biological neural systems, spiking neural networks (SNNs) are considered suitable for adding intrinsic noise for probabilistic computing on hardware level. Hardware implementation using non-von Neumann architecture of SNN-based complementary metal–oxide–semiconductor (CMOS) technology is proved to be energy-efficient and scalable with high computing speed (Merolla et al., 2014), owing to the mature manufacturing technology of metal–oxide–semiconductor field-effect transistors (MOSFETs). However, both the size and energy scaling of Si-based MOSFETs confront new challenges owing to the limitations imposed by the quantum mechanics of materials (Frank et al., 2001). Therefore, novel materials and devices are required to satisfy the rapidly growing demand of energy efficiency and feature size. Emerging electronic devices, such as memristors, CMOS compatible ferroelectric field-effect transistors (FeFETs), and electrolyte-gated transistors, have proved their capability of mimicking the synaptic plasticity based on the controllable conductance under electrical stimulus (Wang and Zhuge, 2019; Choi et al., 2020; Zhu et al., 2020). Although researchers attempted to control the random nature of these emerging devices in certain deterministic fields, such as non-volatile memory, the unpredictable random dynamics have been proved its contribution to sever as the true random number generators (Mulaosmanovic et al., 2018c; Carboni and Ielmini, 2019) and stochastic artificial neurons (SANs) (Parihar et al., 2018; Dang et al., 2019; Deng et al., 2020). In comparison with the CMOS-based neurons, the emerging artificial neurons for probabilistic computing present three advantages, namely circuit simplicity, intrinsic and unpredictable randomness, and reduced feature size. In other words, the dynamically neuronal behavior can be implemented using a simple circuit with several components rather than tens of transistors. Furthermore, the intrinsic and unpredictable randomness renders additional digital circuits unnecessary for generating quasi-stochastic noise. Finally, two-terminal devices can achieve reduced feature size rather than three-terminal transistors using the same CMOS process.

The remainder of this review article is organized as follows. Section “Biological Neuron and Its Conventional Counterpart” introduces the basic microstructure, dynamics of ion exchange, mathematical models, and integrated circuits that represent the dynamics of output spike in biological neurons. Section “Emerging SAN Devices” comprehensively reviews the emerging devices with inherent stochastic features, such as random formation and rupture of conductive filament (CF), random nucleation of domains, casual phase changes in terms of physical mechanisms, and hardware primitives of SANs. In section “Discussion,” we compare and discuss the performances of the traditional silicic and emerging SANs. Finally, section “Summary and Outlook” presents the existing challenges and active trends of stochastic neuromorphic computing algorithms based on emerging devices.

## Biological Neuron and Its Conventional Counterpart

### Biological Neuron and Its Physical Models

**FIGURE 1:**
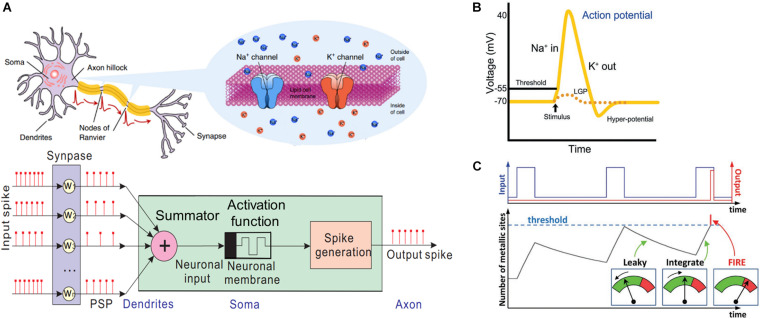
**(A)** Schematic of a biological neuron and an equivalent artificial neuron (Yi et al., 2018). **(B)** Diagram of membrane potential in the Hodgkin–Huxley (HH) model. **(C)** Schematic of the leaky integrate-and-fire (LIF) model (Stoliar et al., 2017).

Most biological neurons comprise dendrites, soma, axon, and a cell membrane separating the inner and outer regions of a neuron, as illustrated in the top panel of Figure 1A. Dendrites connect with the axon of a pre-neuron and receive encoding spikes through a gap, referred to as a synapse, by collecting the chemical neurotransmitters released by the pre-neuron. The potential between the inner and outer regions of the membrane can be tuned and transferred to soma by regulating the Na^+^ and K^+^ concentrations through ion channels. The signals are subsequently summed in the soma. If the local graded potential (LGP) reaches the threshold, an output spike is generated and transferred to a post-neuron through the axon. The bottom panel of Figure 1A illustrates the equivalent schematic of an artificial neuron, which can be divided into three functional components, namely the summator for input spikes, activation function, and output spike generator.

Despite an insufficient understanding of neuron network functions, several models have been proposed, including Hodgkin–Huxley (HH), Morris Lecar (ML), FitzHugh–Nagumo (FHN), integrate-and-fire (IF), and leaky integrate-and-fire (LIF) models, to describe the operation of neurons. The HH model physically describes the dynamics of ion channels in the neuron membrane when an output spike is triggered. Figure 1B depicts the graph of membrane potential vs. time, which can be divided into three identifiable parts, namely the resting period, depolarization, and hyperpolarization. When input spikes from the pre-synapses cause the depolarization of the membrane (LGP) by opening the Na^+^ permeable channels, Na^+^ ions encounter the cell and increase the LGP to a positive potential. If the LGP attains a threshold, K^+^ channels open and allow K^+^ ions to flow out through the cell membrane, resulting in the confinement for continuous rising membrane potential. As the membrane potential increases, a higher number of Na^+^ channels are closed until the maximum potential is attained, whereas the ejected K^+^ ions deplete the potential. Once the membrane potential reaches a certain state, K^+^ channels are consecutively closed to generate the hyperpolarization before returning to the primary state. The ML model is another biophysical neuron model, although certain properties, such as spike-frequency adaptation, are absent.

Although the HH and ML models are biophysically meaningful and measurable, it is difficult to code and analyze data using these models during neuromorphic computing. Hence, several biologically plausible neuron models, such as the FHN, IF, and LIF models were proposed. Although the FHN model does not exhibit the bursting property or chaotic dynamics owing to the lack of refractory time, it is commonly used for neuromorphic computing owing to its simplicity and ability to reproduce various biological behaviors. Additionally, the IF and LIF neuron models use linear equations with a single variable, rendering them the most popular models in computational neuroscience. Figure 1C illustrates the schematic of the membrane potential vs. the input impulses in the LIF artificial neurons. Herein, the input spikes are temporally and spatially integrated to induce the neuron membrane potential increase. When LGP attains the threshold, an action potential is triggered transferring the potential to the post-neuron. Otherwise, LGP leaks and returns to the resting period.

### Conventional Silicon Artificial Neurons

**FIGURE 2:**
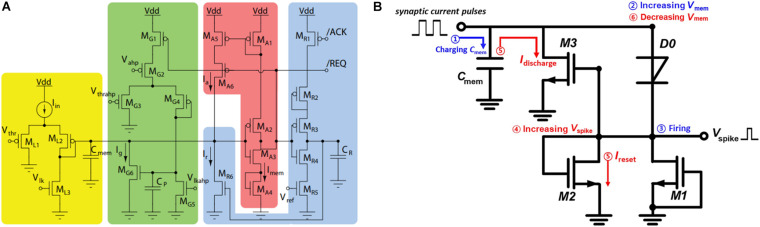
**(A)** Leaky integrate-and-fire (LIF) neuron circuit based on complementary metal–oxide–semiconductor (CMOS) technology (Indiveri et al., 2011). **(B)** Integrate-and-fire (IF) neuron circuit using a *p-n-p-n* diode (Park et al., 2021).

Conventional silicon circuits have been widely used to construct the synapses and neurons in ANNs owing to their mature production technology. Figure 2A illustrates a typical implementation of an artificial neuron with neuromorphic LIF behavior (Indiveri et al., 2011). Herein, the LIF circuit is composed of an input low-pass filter model (yellow), a spike event generator (red), reset block (blue), and spike integration block for spike-frequency adaptation (green). The circuitry is complex with 21 transistors and several capacitors that renders the manufacturing difficult and generates chip-level heat dissipation issues. To reduce the components used in the silicon neuron, an IF neuron circuit using a *p-n-p-n* diode (Park et al., 2021) was proposed (Figure 2B). Herein, the neuron circuit features temporal integration, refractory period, and tunable output spike frequency. Despite the low energy consumption and reduced number of components (three transistors, one diode, and one capacitor), advanced functionalities, such as frequency adaptation and sub-threshold oscillation are absent.

Typically, the aforementioned silicon neurons are used in deterministic neural networks. To achieve inherent stochastic characteristics in the ANN circuits, additional circuits are required to produce quasi-stochastic noises. To further simplify the artificial neuron circuitry, an LIF circuitry (Lim et al., 2015) with threshold switching (TS) components was proposed, which required only three resistors, two capacitors, and two TS memristors to simulate the complete LIF behavior. Moreover, the inherent stochasticity of the emerging devices can form the basis of a new method for constructing true probabilistic neural networks.

## Emerging SAN Devices

The advancements in non-linear electronic devices resulted in the construction of stochastic neuromorphic computing systems with lower energy consumption and limited circuit area. Herein, we systematically introduce certain representative progressed features of artificial neurons with inherent stochasticity, which demonstrate device-to-device (D2D) and cycle-to-cycle (C2C) variations in nature. The discussion includes: (i) filament-based neuron, (ii) ferroelectric neuron, (iii) spintronic neuron, (iv) phase-change neuron, and (v) metal-to-insulator transition (MIT) neuron.

### Filament-Based Neuron

After the memristor was initially proposed (Chua, 1971; Chua and Sung Mo, 1976) and verified (Strukov et al., 2008), it has been increasingly considered for emerging non-volatile random-access memory and brain-inspired neuromorphic computing owing to its simple structure, fast write and read speed, excellent retention time, compatibility with CMOS procedure, and gradual conductance. Various physical mechanisms, such as the valence change mechanism (VCM), electrochemical metallization (ECM), charge trapping/detrapping, and thermochemical reactions in semiconducting metal oxides were introduced to explain the resistive switching phenomenon. Several researchers presented detailed explanations of these mechanisms (Pan et al., 2014; Lee J. S. et al., 2015). Among them, both VCM and ECM are based on ion migration and corresponding redox reactions. Herein, a CF is formed between the electrodes, and its formation and rupture result in the resistive switching between high-resistance state (HRS) and low-resistance state (LRS). Hence, they can be classified as filament-based memristors. However, ECM and VCM differ in terms of the migrating ions, wherein oxygen vacancy migration results in VCM-type resistive switching, whereas ECM is induced by active metals, such as Ag, Cu, and Ni. Typically, filament-based memristors demonstrate the disordered distribution of SET and RESET voltages owing to the random formation and rupture of CFs. Although the disordered parameters of filament-based memristors have been optimized using several feasible approaches (Shi et al., 2011; Li H. Y. et al., 2020; Sun Y. et al., 2020), CF-based memristors face numerous challenges in terms of commercial applications. Nevertheless, this type of natural randomness in CF-based memristors is highly suitable for constructing stochastic neural networks.

A typical filament-based memristor comprises a metal–electrolyte–metal sandwich structure. Generally, compliance current is used to manipulate the filament strength. In the case of strong filaments, non-volatile switching behavior is obtained and the state can be maintained for years. By contrast, weak filaments evoke volatile TS. Furthermore, the conductance of memristors can be tuned to a quantum degree under a proper stimulus. This disorder and gradual conductance render the filament-based memristors inherently appropriate for constructing SANs.

**FIGURE 3:**
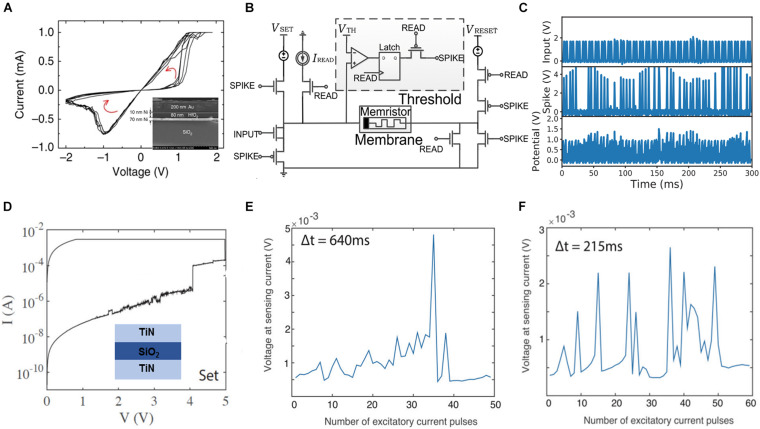
**(A)** Bipolar resistive switching characteristics of a non-volatile memristor. Inset shows the scanning electron microscope images of a cross-section of the memristor. **(B)** Circuitry of artificial neuron equipped with complementary metal–oxide–semiconductor (CMOS) circuit. **(C)** The stochastic output behavior under a certain stimulus. Based on the impulse neuron characteristics of hafnium oxide (HfO_2_) memristor, the neuron response is obtained for an input of V_th_ = 0.7 V at 100 Hz (Wang J. J. et al., 2018). **(D)** An abrupt SET process in I/V sweeps of SiO_x_ resistive random-access memory (RRAM) cells during non-volatile memory switching. Device output in the leaky integrate-and-fire neuronal model with the voltage response measured based on an inductive current pulse of 1 mA immediately after excitation of a 4 mA current pulse at an interval of **(E)** 640 ms and **(F)** 215 ms (Mehonic and Kenyon, 2016).

An effective approach to construct artificial neurons is using the non-volatile memory switching to fulfill the accumulation process of biological neurons, which corresponds to the summator function. This necessitates additional circuits to implement the assessment of threshold membrane potential, spike generator, and a feedback path to reset the memristor to its primary state. A study reported the implementation using a non-volatile memory cell with an Au/Ni/HfO_2_/Ni structure (Wang J. J. et al., 2018). Figure 3A depicts the bipolar resistive switching behavior, wherein the inset illustrates the device structure obtained using a scanning electron microscope. Figure 3B schematically depicts the coupling of memristor with the simplified CMOS circuit. Herein, the memristor integrates the input spikes from pre-synapse, the comparator chip estimates whether the membrane potential exceeds the threshold, and the spike generator chip triggers an output spike after the threshold is attained. Subsequently, the reset chip generates a pulse to achieve the primary state of the LIF neuron. Additionally, a hybrid artificial neuron randomly generates spikes owing to the random resetting event of the memristor under a certain stimulus beyond the threshold voltage. Thus, a stochastic LIF artificial neuron was implemented. Furthermore, the frequency of the output spikes can be tuned by changing the threshold voltage. The maximum output frequency reaches up to 100 kHz, as illustrated in Figure 3C. Figure 3D depicts another implementation of an LIF neuron based on non-volatile memory, wherein the inset represents the SET process and schematic structure. The probability of firing can be tuned based on the interval of input impulses. Figures 3E,F depict the output spikes under excitatory input current pulses (4 mA). Herein, we observed that smaller the interval, lesser is the number of pulses required to trigger output spikes.

**FIGURE 4:**
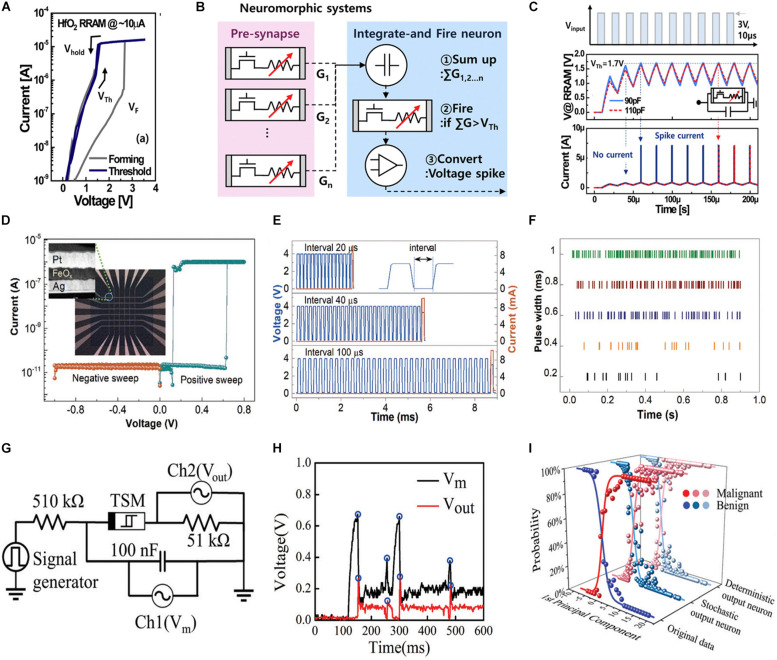
**(A)** Threshold switching (TS) characteristics of the valence change mechanism (VCM) cell at a compliance current of 10 μA. **(B)** Schematic of the neuromorphic system comprising neurons and synapse elements. **(C)** Output dynamics of VCM-based artificial neuron (Woo et al., 2017). **(D)** Volatile-switching behavior of Pt/FeO_3_/Ag device. Inset shows multiple cells with cross-bar structure and the cross-section view of one device under high-resolution transmission electron microscopy inspection. **(E)** Dependence of integrate-and-fire on the input pulse interval. **(F)** Raster plot of firing patterns with respect to time for a memristive neuron with 10 MΩ in series on a dedicated circuit board (Zhang et al., 2018). **(G)** Schematic of a neuronal circuit with the input voltage pulses originating from the signal generator. **(H)** Measured stochastic spike events of the CuS/GeSe-based neuronal circuit. **(I)** Comparison of the output probability of deterministic and stochastic neurons (Wang et al., 2021).

Another appealing approach of constructing artificial neurons is to use the TS device, which can mimic the summator behavior of a biological neuron. Moreover, the volatile nature of TS renders the reset and spike generator circuits unnecessary. Previously, a simple hardware implementation of SNN using VCM devices was accomplished (Woo et al., 2017). Herein, a non-volatile memory based on TiN/HfO_2_/Ti/TiN was used as a synapse, whereas the dynamic neuron behavior was fulfilled by a TS device (Figure 4A). Figure 4B illustrates the schematic of the SNN. The TS device was coupled with a transistor in series and a capacitor in parallel. No output current spike was initially detected until the capacitor, which serves as the summator, was completely charged (Figure 4C). The TS device determines whether the output voltage spike converted from the current spike must be transmitted via an operational amplifier. Moreover, the leaky behavior was achieved only before the first output spike and the refractory period was absent. Another study reported an ECM-based TS neuron implemented using an Ag/SiO_2_/Pt structure (Zhang et al., 2018; Figure 4D). Figure 4E depicts the completely functional LIF artificial neuron obtained by connecting a load resistor. As the value of neuron membrane potential was estimated using the threshold voltage, additional threshold sensing circuits based on capacitor are not required. The single TS-based artificial neuron can trigger output spikes automatically. The firing rate can be tuned by the interval and width of input pulses, whereas the refractory time relies on the input voltage. Furthermore, the inherent random formation and rupture of Ag CF affected the output spike rate. As depicted in Figure 4F, tuning the input pulse width can generate stochastic outputs with different firing rates. Another implementation of a Cu filament-based TS device (Wang et al., 2021) presented LIF neuron behavior by coupling the device with two resistors in series and a capacitor in parallel (Figure 4G). The capacitor imitates the membrane potential, whereas the resistors limit the total current intensity and divide the input voltage. Figure 4H depicts the measured stochastic spike events of the CuS/GeSe-based neuronal circuit. Based on the firing probability, an uncertain stochastic artificial network with probabilistic inference was finally implemented. After unsupervised deep learning of breast cancer data, the results revealed that the recognition accuracy rating of stochastic neurons is substantially better than that of conventional deterministic neurons, particularly at the overlap area of benign and malignant cancers (Figure 4I). However, the generation of sneak current is an issue in the cross-bar architecture of memristors. To address this, memristors with self-rectified behavior is one of the solutions. A artificial neuron based schottky barrier was implemented (Dang et al., 2019). Herein, the formation and diffusion of Cu-based CF dominate the stochastic output spikes. Additionally, the firing rate of the stochastic neuron relies on the amplitudes of input pulses.

**FIGURE 5:**
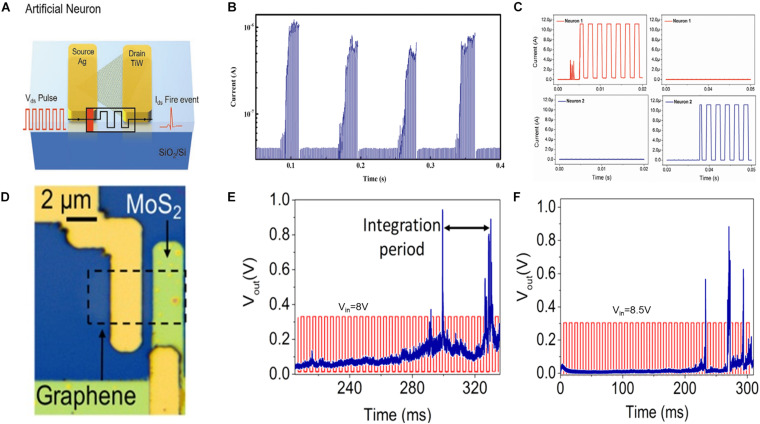
**(A)** Schematic of a planar artificial neuron with an Ag/MoS_2_/TiW structure. **(B)** Realization of leaky integrate-and-fire behavior of MoS_2_-based memristive device under a continuous pulse train. **(C)** The classification result based on the presence and absence of neuron firing (Hao et al., 2020). **(D)** Optical image of the MoS_2_/graphene threshold switching (TS) device. Output spikes under an impulse train with amplitude modulation at **(E)** 8 V and **(F)** 8.5 V. The integration time, refractory period, and stochastic output behavior are obtained (Kalita et al., 2019).

The emerging two-dimensional (2D) materials, such as graphene oxide (Wang et al., 2017), WS_2_ (Kumar et al., 2019), and MoS_2_ (Li et al., 2018), are promising candidates for constructing energy-efficient memristors owing to their advantages in terms of thickness and high metal ion mobility. A study implemented (Hao et al., 2020) a planar memristive device with the structure of Ag/monolayer MoS_2_/TiW (Figure 5A). The distance between the two electrodes is essential for tuning the property of a memristor because the device exhibits volatility only when the distance is greater than 500 nm. Figure 5B illustrates the realization of the LIF behavior of an MoS_2_-based memristive device under a continuous pulse train without an auxiliary circuit. The obtained simple neuron network implemented the image classification function by connecting four memristive synapses. The images are encoded into the pulse train, input into the synapses, and the firing event reveals the classification result (Figure 5C). Another approach (Dev et al., 2020) of obtaining an energy-efficient device is to construct a vertical structure using monolayer MoS_2_. The stochastic LIF behavior can be achieved by operating the TS device at 0.3 V and maintaining an endurance of up to 5 × 10^6^ cycles. Moreover, graphene is used as the inert electrode to further reduce the thickness of the TS device (Kalita et al., 2019). Figure 5D depicts the optical image of the memristor device. Based on the formation and rupture of Ni-based CF, this graphene/MoS_2_/Ni neuron can stochastically generate output spikes with LIF dynamics. Figures 5E,F illustrate the pulse amplitude-modulated frequency response. As indicated in the figures, increasing the input pulse amplitude can effectively increase the firing probability. Interestingly, the refractory period was obtained owing to the diffusion of CF.

**FIGURE 6:**
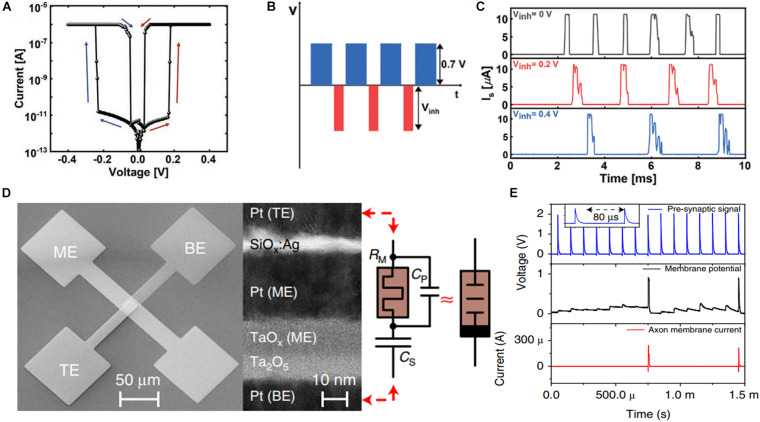
**(A)** Ovonic threshold switching (TS) behavior in Ag/HfO_2_/Ag device. **(B)** Schematic of the input pulse program to verify the property of inhibitory postsynaptic potential (IPSP). **(C)** Firing rate modulation based on the inhibitory pulse amplitude in the opposite direction (Kim T. et al., 2020). **(D)** Scanning electron micrograph of the plane view of the integrated dynamic pseudo-memcapacitor and a transmission electron micrograph of the cross-section. **(E)** Dynamics of the neuro-transistor integrate-and-fire process, which exhibits the input pulse train (top panel), membrane potential (middle panel), and output spike sequence of axon membrane current (bottom panel) (Wang Z. et al., 2018).

Although unidirectional TS devices can mimic the dynamic neuron behavior only under the excitatory stimulus, the inhibitory stimulus is essential in the human brain neuron system. Therefore, ovonic TS devices were developed, which initiated an unprecedented path of using both stimuli simultaneously (Kim T. et al., 2020). Figure 6A illustrates the electrical property of a prototypical LIF neuron based on the Ag/HfO_x_/Ag device. After optimized annealing using N_2_, a high on/off ratio of approximately 6 × 10^7^, low threshold voltage of 0.19 V, low variability of 0.014, and endurance of over 10^6^ cycles were achieved. The LIF neuron behavior under both polarity of applied voltage, namely the excitatory and inhibitory stimuli, was obtained by connecting a capacitor in parallel. Figure 6B illustrates the schematic impulse stimulus train. The firing rate can be modulated based on the inhibitory pulse amplitude in the opposite direction, as depicted in Figure 6C. This progress verified the role of the inhibitory postsynaptic potential property in a single artificial neuron and the feasibility of the synaptic weight change through the bipolar TS device. Figure 6D depicts another implementation of an artificial neuron based on an ovonic TS device. As indicated in the figure, connecting a capacitor in series and a MOSFET on the gate renders the artificial neuron capable of handling spatial and temporal pre-synaptic spikes. Figure 6E illustrates the dynamics of the neuro-transistor IF process. Regardless of the deterministic output spikes obtained from the aforementioned studies, stochastic computing based on ovonic TS neurons remains a suitable choice owing to its ability of simultaneously withstanding excitatory and inhibitory stimuli in a single neuron.

### Ferroelectric Neuron

Ferroelectric materials were discovered nearly a century ago (Valasek, 1921) and used to develop ferroelectric RAM (FeRAM) owing to their spontaneous polarization (Scott and Paz de Araujo, 1989). In the late twentieth century, the development of thin-film growth technology confined the thickness of the ferroelectric films to 100 nm. Consequently, the progression of ferroelectric-based devices was delayed for nearly 30 years. In recent years, the development of thin-film deposition technology and the discovery of new ferroelectric materials with CMOS process compatibility led to the fabrication of nanoscale thin films with high crystal quality on a large scale, reviving the investigations on ferroelectric memory devices.

**FIGURE 7:**
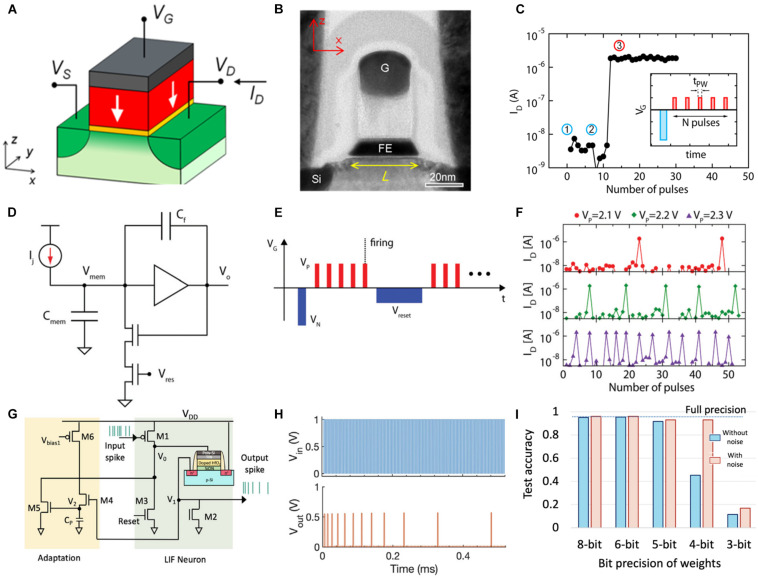
**(A)** Structure of a nanoscale ferroelectric transistor. **(B)** Transmission electron microscopy (TEM) image of a ferroelectric transistor device. **(C)** Accumulative polarization reversal in ferroelectric transistors. The inset depicts the sharp switching from OFF to ON after receiving several identical impulses. **(D)** Schematic of an axon-hillock complementary metal–oxide–semiconductor (CMOS) neuron. **(E)** Pulsing scheme for implementing an integrate-and-fire (IF) cycle and an arbitrary refractory period, after which a new IF cycle begins (Mulaosmanovic et al., 2018a). **(F)** Consecutively repeated IF cycles for different values of *V*_*P*_. **(G)** Circuit implementation of a ferroelectric field-effect transistor (FeFET)-based spiking neuron. The leaky integrate-and-fire (LIF) neuron is implemented using one FeFET and three transistors (M1–M3). Biologically inspired homeostatic plasticity is implemented using additional transistors (M4–M6). **(H)** The decreasing output spike frequency exhibiting spike-frequency adaptation. **(I)** Comparison of test accuracies for different bit precisions of weights with and without noise (Dutta et al., 2020).

Hafnium oxide (HfO_2_) is one of the most popular materials with CMOS compatibility as they exhibit ferroelectric property at a thickness of less than 10 nm (Böscke et al., 2011). Moreover, stochastic nucleation of the ferroelectric domain was discovered in HfO_2_-based FeFET (Mulaosmanovic et al., 2017; Alessandri et al., 2018). Typically, the stochastic domain nucleation occurs in the proximity of its coercive electric field (Shin et al., 2007), whereas that in HfO_2_ can occur in sub-coercive electric field regions, indicating the potential multilevel resistance states for inference neuromorphic computing. Furthermore, impulse dependence measurement was implemented on the FeFET device with a polysilicon/TiN (8 nm)/Si:HfO_2_ (10 nm)/SiON (1.2 nm) gate stack (Mulaosmanovic et al., 2018b). Figures 7A,B depict the schematic structure and transmission electron microscopy image of a nanoscale ferroelectric transistor, respectively. Typically, the domain in ferroelectric devices can be reversed using a single pulse. Additionally, the FeFET demonstrates binary storage owing to the ferroelectric polarization switching (polarization-up and polarization-down). In this case, sharp switching from HRS to LRS occurs only after several identical pulse stimuli with a pulse amplitude of 2.2 V and pulse width of 1 μs are generated, as shown in Figure 7C. Ferroelectric domains near the grain boundary are considered to have a lower coercive field than that within the grain. Initially, domains close to the grain boundary reverse under impulse stimulus, and the polarization orientation inside the grain subsequently undergoes reversal owing to the continuous application of impulses. This is similar to the integration behavior in neurons. When the polarization reversal accumulates to a certain extent, the polarization orientation reverses on a macroscale, increasing the channel current. If the current attains the threshold when a CMOS auxiliary circuit is connected, the firing of impulses is initiated (Figure 7D). Subsequently, the HfO_2_-based FeFET cell resets to the original state using a reset circuit and awaits the firing of the next impulse. The aforementioned process is the typical LIF behavior in artificial neurons. Figure 7E illustrates the pulsing scheme for implementing an LIF cycle and Figure 7F depicts the repeated impulses of IF cycles with different pulse amplitudes. The probability of firing can be manipulated using the amplitude of the applied pulse. The implementation of FeFET-based artificial neurons depletes the traditional CMOS neuron components. However, a comparator circuit can reduce energy consumption. Huang‘s group (Chen et al., 2019) successfully implemented a completely functional LIF neuron using a partially crystallized Hf_0.5_Zr_0.5_O_2_ (HZO) layer-based FeFET and a resistor rather than the large capacitor and six transistors in CMOS neurons. Furthermore, they implemented the spike-frequency adaptation function. Owing to the dominant accumulation effect of the ferroelectric layer, the time interval of firing spikes increased during the firing of the initial few spikes until the polarization degradation reduced the accumulation effect. Both excitatory and inhibitory inputs were connected to the HZO-based LIF neuron by connecting a resistor and FET in series to obtain stochastic output signals (Luo et al., 2019). Additionally, SNNs completely based on HZO were accomplished recently (Dutta et al., 2020). Manipulating the cumulative effect of polarization renders the HZO-based FeFET as artificial synapses and LIF neurons. Figure 7G depicts the circuitry of the FeFET-based SAN. Herein, the output spike frequency decreases under a continuous pulse train, indicating the frequency adaptation behavior (Figure 7H). Furthermore, supervised learning on an Modified National Institute of Standards and Technology (MNIST) dataset was performed using a three-layered SNN. The final image recognition accuracy was approximately 95.4%, which was equivalent to that obtained from software simulation. Using the Bayesian hyperparameter optimization approach, stochastic noise induced by the random nucleation of ferroelectric devices was employed to impact the recognition accuracy. Figure 7I illustrates the comparison of test accuracies with and without noise. As indicated in the figure, a stochastic SNN with inference can aid in improving the classification accuracy, particularly at the 4-bit weight.

### Spintronic Neuron

**FIGURE 8:**
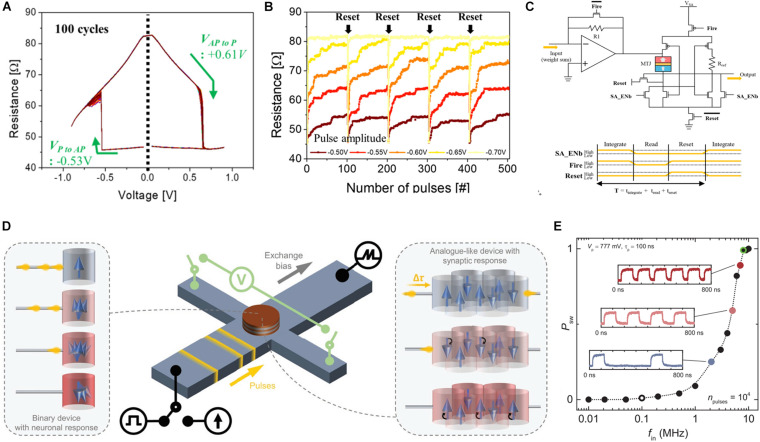
**(A)** Resistance vs. voltage curve in the STT–MTJ device. **(B)** Repeated integration characteristic of the STT–MTJ. **(C)** Neuron circuit and integrate-and-fire behavior (Kim D. W. et al., 2020). **(D)** Functioning of the artificial neuron and synapse based on antiferromagnetic SOT–MTJ. **(E)** Dependence of switching probability on the frequency of incoming pulse trains (Kurenkov et al., 2019).

The prediction of the spin-transfer torque effect (Berger, 1996; Slonczewski, 1996) led to the manipulation of the magnetization state of ferromagnetic materials with electrical current and systematic investigations of spintronic devices. Magne tic tunneling junction (MTJ) composed of two metallic ferromagnetic layers and a tunnel oxide layer is a basic cell of spintronic devices. The thicker ferromagnetic layer with pinned spin polarization is referred to as the pinned layer (PL) or reference layer, whereas the thinner ferromagnetic layer is called the free layer (FL) as its magnetization direction can be altered by the injection of current. When the magnetization direction of the FL is parallel to that of the PL, electrons with the corresponding spin orientation conveniently pass through the tunnel layer, and the device exhibits LRS. Conversely, when the FL is anti-parallel to the PL, the device exhibits HRS. This phenomenon is referred to as the tunnel magnetoresistance effect (Fong et al., 2016). Typically, the spin directions of electrons in ferromagnets are spin-up and spin-down. While the spin electrons matching the direction of the magnetic field can pass through the ferromagnet efficiently, other spin electrons are reflected owing to the momentum conservation. This phenomenon is called the spin filter effect (SFE). When the injected electrons flow from PL to FL, the direction of the magnetic polarization in FL adjusts itself to be identical to that of the PL owing to the corresponding spin electrons. Consequently, the MTJ cell exhibits LRS. By contrast, when the external electric field drives spin electrons from FL to PL, the matched spin electrons pass through the PL and unmatched spin electrons bounce back to the FL owing to the SFE, resulting in the opposite magnetic field orientation of FL and PL. This phenomenon is referred to as spin-transfer torque (STT). Subsequently, the resistance of the MTJ cell changes from LRS to HRS. Hence, the MTJ device is considered to possess bipolar binary memory. Additionally, the irregular magnetic domain and thermal noise result in the stochastic domain reversal (Devolder et al., 2008), rendering the device suitable for probabilistic computing. An artificial neuron was developed with a structure of (W/TiN) electrode/Ta/Pt/(Co/Pt)_6_/Co/Ru/(Co/Pt)_3_/Co/W/Co_2_Fe_6_B_2_ PL/MgO tunnel layer/Fe(Co_2_Fe_6_B_2_) FL/W/Co_2_Fe_6_B_2_ FL/MgO capping/W/(Ta/Ru) electrode (Kondo et al., 2018). The IF function was successfully mimicked using an auxiliary reset circuit, wherein the pulse width was 400 μs and amplitude was 1.15 V at an assistant magnetic field of 750 Oe. Initially, the magnetic domain at the grain boundary reversed owing to the energy barrier at the grain boundary of the MgO layer being lower than that observed inside the grain. Subsequently, continuous impulse stimulus reversed the magnetic domain within the grain achieving the integration behavior. When all domains in FL were anti-parallel to PL, the MTJ cell realized the firing function. Figure 8A depicts the resistance vs. voltage curve of another implementation of an STT–MTJ neuron (Kim D. W. et al., 2020). Herein, the integration and reset processes of membrane potential exhibit excellent endurance, as illustrated in Figure 8B. Figure 8C depicts the basic neuronal IF behavior achieved after coupling the device with appropriate CMOS circuits to reset the MTJ cell. Additionally, the dependence of the integration behavior on the input spike number and amplitude were investigated further. When the amplitude of the input stimulus increased from -0.5 to -0.7 V, no integration behavior was observed. Furthermore, the pattern recognition accuracy of a neural network constructed using an IGZO-based artificial synapse was approximately 76% owing to the lack of proper learning algorithms to train the MTJ-based neural network.

Another approach to construct a magnetic neuron device involves using a spin–orbit torque (SOT)-cell, which is composed of one non-magnet (antiferromagnet) layer, one ferromagnet layer with in-plane magnetization, and a heavy metal electrode. Owing to the spin Hall effect and Rashba effect (Mihai Miron et al., 2010; Shim et al., 2017), the SOT-cell exhibits stochastic magnetization switching. This method has been theoretically proposed to be applied to probabilistic neural computing (Biswas et al., 2015; Sengupta et al., 2015, 2016, 2018) and experimentally proved to be feasible (Ostwal et al., 2018). The probability of domain switching increases with the increasing SOT current amplitude but independent of its polarity. Artificial synapse and neuron have been implemented with a stack of Ta (3 nm)/Pt (2.5 nm)/ Pt_3__8_Mn_62_ (9.5 nm)/Pt (0.6 nm)/[Co(0.3 nm)/Ni(0.6 nm)]_2_/Co (0.3 nm)/MgO (1 nm)/Ru (1 nm) and hall channel of Ta/Pt/PtMn layer for SOT switching (Kurenkov et al., 2019), which equipped the antiferromagnet for the construction of SOT–MTJ. Figure 8D depicts the dynamics of artificial MTJ-based synapse and neuron. Initially, multilevel states were achieved by adjusting the width of impulses from 1 s to 1 ns, and the spike-timing-dependent plasticity function of the artificial synapse was repeatedly measured. A CMOS circuit was used to compare the threshold and trigger the firing action owing to the non-volatile property of the device. Additionally, a pulse train with a width of 100 ns was used to stimulate the SOT–MTJ-based neuron. Figure 8E illustrates the firing (domain reversal) probability as a function of the input frequency. The response frequency of the input pulse reached up to 80 MHz.

In comparison with binary memory devices, memory devices with multiple states are more important for neural computing. Ideally, current-induced domain wall motion in the direction of electron flow is expected to address the bottleneck of MTJ-based neurons (Sharad et al., 2012, 2013). Additionally, multilevel resistance states have been experimentally achieved in an STT–MTJ device (Lequeux et al., 2016), which was realized using pinned domains caused by continuous domain switching. This type of artificial synapse can be naturally coupled to either CMOS neurons or other artificial neurons to implement the firing behavior. Furthermore, other simulation approaches have been utilized to control the wall motion (Hassan et al., 2018; Azam et al., 2020). Although manipulating the nanosized skyrmion can yield multilevel states in ferromagnets (Azam et al., 2018; Chen et al., 2018; Liang et al., 2020), artificial synapses or neural components based on skyrmion have not been reported thus far.

### Phase-Change Neuron

Phase-change materials are a series of alloys that can reversibly transform between amorphous and crystalline states with different optical and electrical transport properties. Typically, the composition includes a ternary alloy of Ge, Sb, and Te, such as Ge_2_Sb_2_Te_5_ (GST). Initially, the incubation of crystal growth occurs inside the amorphous region owing to the application of a low yet wide voltage pulse and Joule heat. Subsequently, the nanocrystals gradually grow until the entire amorphous region transforms into a polycrystalline region. During this process, the resistance of a phase-change memory (PCM) cell changes from HRS to LRS. Conversely, when a short yet high voltage pulse is applied, certain sections in the polycrystalline region melt and cool down rapidly, resulting in an amorphous region. The resistance of the PCM cell transforms from LRS to HRS, indicating the reset process. Additionally, C2C and D2D variations resulting from the random crystal nucleation and the position of Poole–Frenkel sites for carrier transport in the amorphous region render the PCM a key enabling technology for stochastic neural computing.

**FIGURE 9:**
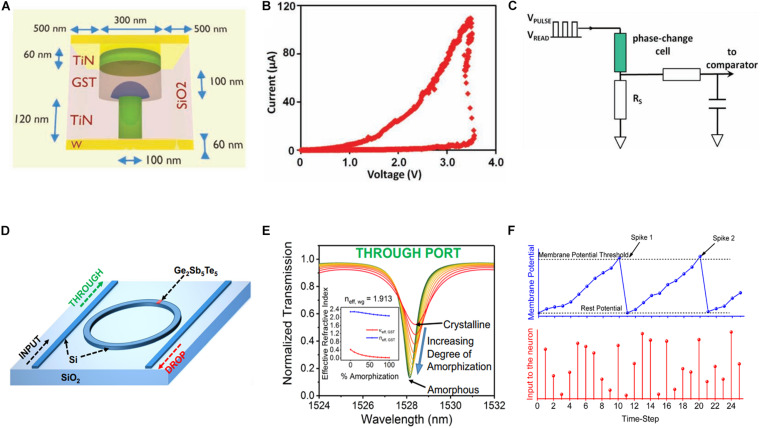
**(A)** Schematic of the phase-change memory (PCM) mushroom-type cell. **(B)** Experimental I–V curve exhibiting a threshold switching voltage from amorphous to crystalline state. **(C)** Phase-change integrate-and-fire (IF) neuron circuit based on a single phase-change cell (Wright et al., 2012). **(D)** An optical neuron based on a GST cell. **(E)** Gradual change of transmission owing to different degrees of amorphization of GST ranging from 0% (crystalline) to 100% (amorphous). **(F)** Behavior of the proposed IF neuron in the spiking neural network (SNN) exhibiting the variation of the membrane potential under the action of incident pulses, thus resulting in the IF action (Chakraborty et al., 2018).

Figure 9A depicts a typical PCM cell comprising a top electrode, a pillar-shaped bottom electrode, confined Joule heating induced by current, and a phase-change material with a hemispherical amorphous region (Wright et al., 2012). Figure 9B illustrates the corresponding typical resistive switching characteristics. Figure 9C depicts a simple firing circuit that aids in realizing the LIF behavior (Tuma et al., 2016). This circuit can mimic the generation of output spikes for the postsynaptic neuron. However, the PCM cell remains in the LRS owing to the non-volatile storage. Based on this phenomenon, a spiking neuron auxiliary circuit with a self-resetting function was proposed (Cobley et al., 2018). Herein, automatic post-spiking resetting was achieved by adding a feedback reset path. After attaining a conductance threshold, output spikes were fired and the phase-change device automatically reset to the initial state, awaiting the next firing. Both the hardware implementation and corresponding algorithms of a PCM-based neural network are equally important. Two PCM neurons were proposed to implement a backpropagation algorithm for hardware neural networks (Li C. et al., 2020). Herein, the forward propagation and backpropagation signals are stored in one PCM cell each, eliminating the requirement of additional memory units. The experiment verified that the total computing area can be reduced to increase energy consumption efficiency.

Owing to the highly contrasting optical properties in the amorphous and crystalline states, PCM is generally used for optical devices. A recent report (Stegmaier et al., 2017) indicated that PCM cells exhibit sub-ns “write” speeds under photonic laser pulse stimuli. Typically, the PCM cell can be heated using the applied laser pulses and transform from an amorphous state with low optical transmission to a crystalline state with high optical transmission. Figure 9D depicts a microring resonator that can be added to obtain an all-photonic phase-change spiking neuron (Chakraborty et al., 2018). The phase-change material can partially absorb the laser wave passing through the microring resonator and its temperature increases owing to the low thermal conductivity (Lyeo et al., 2006). Therefore, when the temperature in the corresponding region attains the melting point (Sebastian et al., 2014), the crystal nucleation occurs in the amorphous region. Subsequently, the optical transmission of the GST cell gradually changes (Figure 9E), which is equivalent to the electric conductance evolution in traditional neural systems. Figure 9F depicts the IF action in the simulated SNN. These simulation results predict that the writing time can be as low as 200 ps with an average energy of 4 pJ in a “write” step. Further research on optical spiking neural networks (Feldmann et al., 2019) determined that increasing the input optical energy at a fixed wavelength initiates the activation function in the output transmission, which can be used to define the firing action. A feedback path was introduced to reset the GST cell to its primary state. Owing to the high bandwidth and fast data transfer rates intrinsic to light, the developed all-photonic neural network can operate several orders of magnitude faster than electrical brain-inspired neural networks, handling large amounts of data in a short time.

### Metal–Insulator Transition Neuron

Unlike the phase-change material, wherein the transition occurs between amorphous and crystalline states, materials based on metal–insulator transition (MIT) can reversibly alter from a crystalline metal to an insulator phase. Both electrons and heat can evoke the transition, and the randomness in nucleation leads to the C2C and D2D difference.

**FIGURE 10:**
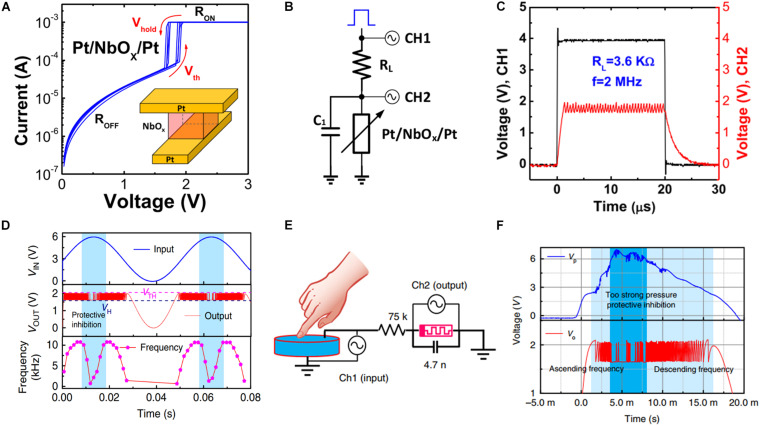
**(A)** Measured I–V threshold switching characteristics of the Pt/NbO_x_/Pt device. Inset depicts the schematic of the fabricated Pt/NbO_x_/Pt device. **(B)** Circuit configuration of an oscillating neuron node with the Pt/NbO_x_/Pt device and a load tunable resistor as a synapse. **(C)** Oscillation characteristics when the load resistor is 3.6 kΩ and output frequency is 2 MHz (Gao et al., 2017). **(D)** Dependence of the output frequency on the input voltage amplitude. **(E)** Schematic of the artificial spiking mechanoreceptor system. **(F)** Dependence of the output spike frequency on the pressure. When the pressure is extremely high, frequency adaptation action protects the device (Zhang et al., 2020).

Various materials, such as VO_2_ (Choi et al., 1996), TiO_x_ (Lee D. et al., 2015), NbO_x_ (Kumar et al., 2017b), SmNiO_3_ (Ha et al., 2011), and compounds such as AM_4_Q_8_ (A = Ga, Ge; M = V, Nb, Ta, Mo; Q = S, Se) (Abd-Elmeguid et al., 2004; Pocha et al., 2005) exhibit MIT characteristics. Among these, VO_2_ and NbO_x_ are the most popular materials used for neuromorphic computing. The typical structure of an MIT device is electrode–MIT material–electrode, and the electrical transport exhibits typical volatile behavior (Figure 10A). Figure 10B depicts the simplest neuron circuit, wherein a resistor and a capacitor are connected in series and parallel, respectively, to obtain an oscillator. Typically, the value of resistance of the load resistor exists in between that of the LRS and HRS. When the external voltage is applied, the MIT device is initially set to LRS, which decreases the divided voltage across the MIT device. Once the divided voltage decreases below the hold voltage, the resistance of the MIT device is reset to HRS. By contrast, when the divided voltage surpasses the threshold, the device is set to LRS again. Figure 10C indicates that the output voltage oscillates owing to the repetition of the set and reset process, during which the frequency can be varied by the load resistor (Gao et al., 2017; Woo et al., 2019). Further research indicated that the applied voltage can regulate the output frequency (Zhang et al., 2020), as depicted in Figure 10D. Additionally, a microwave oscillator circuit was proposed (Zhao and Ravichandran, 2019) to generate output oscillation frequencies as high as 3 GHz with energy consumption as low as 15 fJ/spike. Furthermore, the output voltage frequency can be adjusted based on the external pressure by coupling the device with an afferent sensor, such as a piezoelectric device (Figure 10E). Figure 10F illustrates the protective inhibition behavior exhibited by the device when the applied pressure is extremely high. The result indicates the potential applicability of MIT devices in neurorobotics.

**FIGURE 11:**
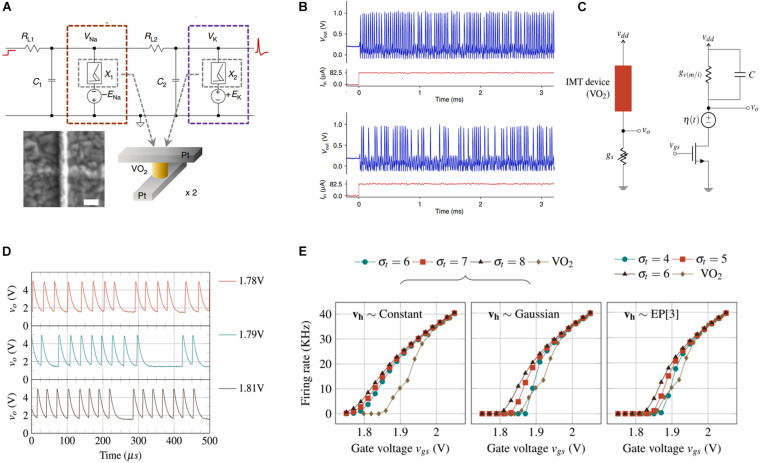
**(A)** Top panel depicts a circuit diagram of a Hodgkin–Huxley (HH) neuron based on two VO_2_ memristors. The bottom panel denotes the device structure. **(B)** Stochastic output spikes under a constant voltage (Yi et al., 2018). **(C)** The left panel represents the VO_2_-based neuron circuit with the VO_2_ device in series with a tunable resistor. The right panel indicates the MIT neuron with the thermal and threshold noises. **(D)** Instantaneous firing under multiple values of *v*_*gs*_. **(E)** Firing rate plotted against *v*_*gs*_ using the analytical and experimental results for different *v*_*h*_ distributions (Parihar et al., 2018).

A completely functional HH neuron circuit (top panel in Figure 11A) was initially proposed using two NbO_x_ oscillating circuits (Pickett et al., 2013) and the extended version included two VO_2_-based memristors (Yi et al., 2018). Herein, each memristor emulates the dynamics of the Na^+^ and K^+^ channels of a biological neuron membrane. When a sub-threshold input is applied, the output membrane potential fluctuates and returns to the initial state, indicating the implementation of the leaky behavior in the HH model. If a super-threshold input voltage is applied, an all-or-nothing spike with a refractory period produces the hyperpolarization potential. A single VO_2_-based active memristor neuron can exhibit the spiking behavior equivalent to that of 23 biological neurons spiking behaviors, which is substantially better than that of the contemporary software deep learning (Izhikevich, 2004). Owing to the random transition between metal and insulator states, the output spikes exhibit stochastic behavior under a certain input impulse (Figure 11B), which is important for the construction of SNN with inference. The left panel in Figure 11C illustrates an FHN neuron circuit with a VO_2_ memristor in series and a tunable resistance (Parihar et al., 2018). Replacing the tunable resistance with a transistor and adding a thermal noise voltage source [η(t)] (right panel of Figure 11C) renders this neuron circuit sufficiently competent to manipulate the random distribution of threshold voltage of the VO_2_-based memristor from both thermal and electrical aspects and control the stochastic firing rate rather than the integration rate (Tuma et al., 2016). Figure 11D illustrates the random output spiking waves obtained from various input voltages. The maximum firing rate can reach up to 30 kHz and energy consumption is 196 pJ/spike owing to the fast transition speed of MIT materials. Figure 11E depicts the firing rate as a function of *v*_*gs*_ based on the introduction of the thermal noise voltage source [η*(t)*]. The experimental results concur with those obtained from the analytical model for Gaussian distribution, validating that the output spikes of VO_2_ neurons demonstrate true stochasticity.

Additionally, chip-level thermal management may face severe challenges if pure VO_2_-based neuron is introduced to the integrated circuit owing to the low Mott transition at approximately 67°C (Chen et al., 2016). This can be mitigated by introducing a dopant that can increase the MIT critical temperature (TC) to approximately 96°C (Krammer et al., 2017). In comparison with VO_2_, NbO_x_ is considered a more suitable option for applications at chip level owing to its higher TC (810°C) (Páez Fajardo et al., 2021).

## Discussion

Table 1 presents a detailed comparison of the hardware implementations of various artificial neurons in terms of the implemented neuron model, support circuit complexity, energy consumption, firing frequency, on/off ratio indicating the capability of synaptic weight accumulation, and advanced functionality. As indicated in the table, traditional CMOS-based artificial neuron is advantageous in terms of energy efficiency owing to the mature processing technology. Conversely, MIT-based artificial neurons can achieve most types of output spike models. Additionally, the complex HH model can be mimicked using only a resistor and a capacitor. Most implementations are bio-mimetic neurons aiming to emulate the basic behavior of biological neurons and require additional hardware, such as resistors and capacitors. However, in comparison with the CMOS-based neurons, the cost of additional hardware in emerging neurons is negligible, aiding the scaling of the overall chip energy, size, and complexity. Moreover, most of the emerging bio-mimetic neurons are two-terminal devices and compatible with CMOS technology, which renders them applicable in different fields.

**TABLE 1:** Comparison between various hardware implementations of artificial neurons.

**Type**	**CMOS**	**Filament**	**Ferroelectric**	**MTJ**	**PCM**	**MIT**
Neuron model	ML	LIF	LIF	LIF	LIF	HH
Material	Silicon	Pt/HfAlO_x_/TiN/Ag/Pt	HfO_2_	Antiferromagnetic SOT^1^	GST	VO_2_
Complexity	5T1C	2R1C	6T	Digital circuit	Digital circuit	1R1C
Energy/spike	2 fJ	16 fJ	1–10 pJ	N/A	5 pJ	5.6 fJ
Firing rate	15.6 kHz	N/A	50 kHz	Tens of kHz	35–40 kHz	3 GHz
On/off Ratio	N/A	10^6^	10^2^	<10	≈10^2^	10^2^
Stochasticity	N/A	Yes	Yes	Yes	Yes	Yes
Other functionalities	Stochastic resonance	Tunable frequency	Frequency adaptation Excitatory and inhibitory stimuli	Ultrahigh frequency response	Tunable frequency	23 spiking behaviors
	Danneville et al., 2019	Lu et al., 2020	Dutta et al., 2020	Kurenkov et al., 2019	Tuma et al., 2016	Yi et al., 2018

*T, R, and C denote transistor, resistor, and capacitor, respectively. ^*a*^Details of the material: Ta (3 nm)/Pt (2.5)/Pt_3__8_Mn_62_ (9.5)/Pt (0.6)/[Co (0.3)/Ni (0.6)]_2_/Co (0.3)/MgO (1)/ Ru (1)/Ta/Pt/ PtMn*.

Although the aforementioned artificial neurons exhibit stochastic neuronal functions, the unique advantages and disadvantages of each emerging stochastic neuron must be addressed. For instance, the endurance of over 10^15^ of an MTJ cell is outstanding. However, the major challenge for MTJ-based neurons is constructing ultrahigh-density networks using complex processing units (Grollier et al., 2016). Additionally, the tunneling magnetoresistance ratio of MTJ cells is experimentally determined to be 600% to date (Ikeda et al., 2008), which implies that a higher number of neuron cells are required to integrate the pre-synaptic input spikes. Conversely, filament-based neurons exhibit on/off ratios, accessible endurance cycles, low operating voltage, and adequate energy efficiency. However, the volatile memory in most cases can respond only in one direction of the input stimulus, such as the excitatory postsynaptic potential. Additionally, further optimizations of fabrication processes are inevitable for ovonic TS. As the electrical field required for the oxygen ions to escape the lattice is 10 MV/cm (Wong et al., 2012) and that of Ag^+^ to diffuse in SiO_2_ is less than 1 MV/cm (Waser and Aono, 2007; Yang et al., 2013), ECM-based devices are suitable for constructing artificial neurons with low power dissipation. Moreover, the inherent mechanism of TS relaxation caused by the dissolution of metal particles renders the relaxation time of an ECM cell large and restricts the output spike frequency (Lee et al., 2019). Although the TS in MIT devices is ultrafast (up to several nanoseconds) in terms of switching speed, the on/off ratio is generally less than 10^2^. Moreover, the range of the synaptic weighted sum can be restrictive, resulting in the requirement of numerous neurons to integrate the input spikes. Nevertheless, MIT-based neurons can mimic most biological spiking models (Yi et al., 2018). Similar to MIT-based neurons, PCM neurons demonstrate ultrafast switching speed, excellent endurance, high energy efficiency, and scaling down characteristics. However, additional spike generator and feedback circuits are required to trigger the output spikes and reset the device to the initial state, respectively, after the non-volatile inherence causes the firing action. Furthermore, the complexity and size of auxiliary circuits should be scaled down. FeFET-based neuron demonstrates adequate energy efficiency, high output spike frequency, and responds to both excitatory and inhibitory stimuli in a single cell. However, FeFET-based artificial neurons are three-terminal devices that need to be scaled down further. Moreover, the reported FeFET-based neuron with self-resetting and automatic firing functions was equipped with a partially crystalline ferroelectric thin film, rendering it difficult to establish a standard fabrication procedure of ferroelectric films.

Based on the aforementioned discussion and taking predictions about technology scaling of the next decade into account, one can collect a number of requirements for SANs: (i) Stochastic output. The frequency distribution of the output (spiking) should be random and unpredictable. (ii) Endurance. The emerging neurons must exhibit a high endurance over 10^6^ cycles as the spiking algorithms rely on a continuous operation procedure. (iii) On/off resistance ratio. To reduce the quantity of SANs used in SNN and decrease the total energy consumption, a high on/off resistance ratio (10^3^) can improve the capacity of summing the weights from potentiated and depressed synapses. (iv) Energetic efficiency. Though the accuracy of SNN is not extremely high comparing to the ANN, SNN may take an important role in sensors or embedded systems, thus requiring low-power consumption. Energy consumption per spike should be as low as possible to maintain the functionality for long times even in battery-operated devices. (v) Automatic reset. Stochastic neurons need to automatically reset to their primary states after each IF cycle. Hence, the accessory circuit to reset the neurons is needless, which is beneficial to the chip size and energy efficiency.

## Summary and Outlook

SANs that can effectively mimic the sources of background noise with true stochasticity are essential components in SNNs when used for probabilistic computing. Although the emerging artificial neurons can imitate the basic functionalities, such as the all-or-nothing firing, refractory period, tunable output frequency, and frequency adaptation, they cannot mimic advanced functionalities of an actual biological neuron, such as lateral inhibition, variable spiking modes, and chaos. Further analyses are required to ensure that the artificial neurons are more bio-mimetic, which warrants dedicated investigations on device dynamics.

Although the hardware primitives of SANs are at the early stages of development, the corresponding training algorithms should be developed considering the future applications of randomness in computation. Appropriate algorithms can exploit the potential of unique characteristics in the emerging SANs to attain high computational efficiency, low power consumption, and maintain dynamic artificial neurons.

Comprehensive simulation of the inference functionality in SNN requires a close collaboration of different fields, such as biological neuroscience, material science, microelectronics engineering, and computational neuroscience. Biological scientists must reveal the operation and functionality of a human brain for the benefit of researchers in other fields and lay the foundation for constructing multi-functional and universal artificial intelligence systems. This close collaboration of scientists from various fields can significantly enhance the accuracy of SNNs.

## Author Contributions

FZ proposed and guided the direction of the manuscript. Z-xL wrote the main body of this article. X-yG collected the literatures and drew the figures and captions. JW wrote the abstract and summary. All authors contributed to discussions regarding the manuscript.

## Conflict of Interest

The authors declare that the research was conducted in the absence of any commercial or financial relationships that could be construed as a potential conflict of interest.

## Publisher’s Note

All claims expressed in this article are solely those of the authors and do not necessarily represent those of their affiliated organizations, or those of the publisher, the editors and the reviewers. Any product that may be evaluated in this article, or claim that may be made by its manufacturer, is not guaranteed or endorsed by the publisher.
